# Steel Wire Mesh as a Thermally Resistant SERS Substrate

**DOI:** 10.3390/nano8090663

**Published:** 2018-08-26

**Authors:** Tomasz Szymborski, Evelin Witkowska, Krzysztof Niciński, Zuzanna Majka, Tomasz Krehlik, Tomiła Deskur, Katarzyna Winkler, Agnieszka Kamińska

**Affiliations:** 1 Institute of Physical Chemistry, Polish Academy of Sciences, Kasprzaka 44/52, Warsaw 01-224, Poland; tszymborski@gmail.com (T.S.); evelinwitkowska@wp.pl (E.W.); krzysiekn92@gmail.com (K.N.); zuza_majka@wp.pl (Z.M.); tomasz_krehlik@wp.pl (T.K.); tomila@interia.pl (T.D.); kwinkler@ichf.edu.pl (K.W.); 2 Soft Materials Laboratory, Institute of Materials, Ecole Polytechnique Fédérale de Lausanne, 1015 Lausanne, Switzerland

**Keywords:** Surface-enhanced Raman spectroscopy (SERS), wire mesh, steel mesh, SERS platform, *Escherichia coli*, *Bacillus subtilis*

## Abstract

In this paper, we present novel type of Surface-enhanced Raman spectroscopy (SERS) platform, based on stainless steel wire mesh (SSWM) covered with thin silver layer. The stainless steel wire mesh, typically used in chemical engineering industry, is a cheap and versatile substrate for SERS platforms. SSWM consists of multiple steel wires with diameter of tens of micrometers, which gives periodical structure and high stiffness. Moreover, stainless steel provides great resistance towards organic and inorganic solvents and provides excellent heat dissipation. It is worth mentioning that continuous irradiation of the laser beam over the SERS substrate can be a source of significant increase in the local temperature of metallic nanostructures, which can lead to thermal degradation or fragmentation of the adsorbed analyte. Decomposition or fragmentation of the analysed sample usually causea a significant decrease in the intensity of recorded SERS bands, which either leads to false SERS responses or enables the analysis of spectral data. To our knowledge, we have developed for the first time the thermally resistant SERS platform. This type of SERS substrate, termed Ag/SSWM, exhibit high sensitivity (Enhancement Factor (EF) = 10^6^) and reproducibility (Relative Standard Deviation (RSD) of 6.4%) towards detection of *p*-mercaptobenzoic acid (*p-*MBA). Besides, Ag/SSWM allows the specific detection and differentiation between Gram-positive and Gram-negative bacterial species: *Escherichia coli* and *Bacillus subtilis* in label-free and reproducible manner. The unique properties of designed substrate overcome the limitations associated with photo- and thermal degradation of sensitive bacterial samples. Thus, a distinctive SERS analysis of all kinds of chemical and biological samples at high sensitivity and selectivity can be performed on the developed SERS-active substrate.

## 1. Introduction

Surface-enhanced Raman spectroscopy (SERS) is nowadays one of the most powerful and reliable method in a wide range of applications including biochemistry [1], biomedical analysis [2], forensics, bio- and chemical hazards [3] and environmental monitoring [4,5].

In SERS effect, for molecules adsorbed onto metallic nanostructures, the Raman signal is amplified up to 14 orders of magnitude. Two mechanisms, an electromagnetic (EM) and a chemical (CT) are involved in SERS effect. An electromagnetic enhancement results from the amplification of incident light due to excitation of localized surface plasmon resonance (LSPR) of the metal surface. It was found that the morphology and dielectric environment of the plasmonic nanostructures plays a crucial role in the EM mechanism. The chemical enhancement process involves the charge-transfer occurring between the metal surface and adsorbed molecules, which can enhance the transition polarizability of adsorbates [6]. Typically, the electromagnetic enhancement can reach factors 10^3^–10^11^, whilst the chemical enhancement contributes additional factor up to 10^3^ [7,8]. As a result of these mechanisms, SERS ensures the ultratrace detection of analytes down to single molecules [9]. Besides ultrasensitvity and fingerprint specificity, SERS technique offers nondestructive, label–free, and fast detection of analytes under a wide range of conditions (excitation wavelength and power of the laser, temperature, pressure, presence of water).

These features lead to an increase of the practical applications of SERS technique especially in biological materials studies, starting from single macromolecules and ending with whole cells (for example, microorganisms) [10,11,12,13,14]. The SERS spectra of biomolecules carries the information about their structure. This technique also offers the quantitative and qualitative studies with the possibility of multiplexed detection of analytes in complex fluids such as blood, cerebrospinal fluid or urine. 

However, the practical implementation of SERS technique in a clinical setting is hindered by the difficulties in generation of a model SERS–active nanostructures. Such SERS substrate should reveal homogenous and high enhancement factor (EF) across the surface, as well as chemical and physical stability. Additionally, the fabrication method of SERS substrates should be as cheap as possible and provide large-scale production. The commonly used SERS substrates are based on the nanostructured noble metals e.g. Ag, Au, Cu, however the most frequently used metal is silver as it is quite cheap in comparison with gold and provides very high SERS enhancement (much higher than for copper). Interestingly, SPR (surface plasmon resonance) application of Ag is not only important from the point of view of SERS studies, but also covers other technologically important fields such as photocatalysis [15,16].

Today, a variety of techniques have been applied to fabricate SERS nanostructures, such as electrochemical methods [17], nanosphere lithography [18], electron-beam lithography [19], nanoimprinting lithography [20], vapour layer deposition [21], colloidal suspension [22], and many other strategies.

Lately, our research group has developed several types of novel nanomaterials, which can be used in a wide range of biomedical studies [23,24,25,26,27,28]. In recent research works we have investigated the membrane-based SERS-platforms [26,29], where polymer mats covered with gold nanostructures enable filtration of bacteria from fluids (for example, blood plasm), their immobilization on the filter surface, and enhancement of Raman signal. This solution overcomes the problem of transferring bacteria from filter to the SERS platform and potential contamination of the sample, and, at the same time, enables detection of very low concentration of bacteria. Nowadays, there are many different types of materials which are used to produce SERS-active substrates, e.g. ceramics [30,31], silver nanorods [32], mesoporous silica [33], ultrafiltration membrane [34], filter paper [35,36] or metal nanocrystals [37]. Particularly interesting are the polymer-based manufacturing techniques, due to the low cost and ease of production. However, polymer substrates cannot meet the demands of high stiffness of the substrate and high heat transfer (i.e. heat dissipation). Therefore, we have expanded our research by developing the thermally resistant SERS platforms. Continuous irradiation of the laser beam over the SERS substrate can be a source of significant increase in the local temperature of metallic nanostructures, what is a critical issue in SERS measurements of heat-sensitive materials [38,39]. Thermal degradation or fragmentation of the analyte is a vital problem during examination of heat-sensitive materials like DNA, proteins, polymers or lipid bilayers [40]. Moreover, molecules that are adsorbed to the metal surface (e.g. thiols at gold) can desorb at a temperature of 60–100 °C [40]. Decomposition or fragmentation of the analyte often leads to broadening and reduction of the intensity of observed spectral bands, and as a result, to misinterpretation of analytical data [41,42].

Another issue is connected with possible degradation of the polymer in contact with water (biodegradable polymers like poly(L-lactide acid) (PLLA) or polylactid acid (PLA)) or in contact with organic solvents. Biological samples involve water-based samples (blood, urine, cerebrospinal fluid), therefore used polymers should not be biodegradable in water environment, especially when the filtration process is long. 

To conclude, there is a strong need for cheap, versatile, durable, stable SERS-platform which can easily dissipate heat thus can be used with high power lasers. Such platform should, preferably, be resistant to water and other organic and inorganic solvents. In this paper, we present a new type of SERS platforms based on woven wire mesh made of stainless steel (referred here as a stainless-steel wire mesh, SSWM). There are four types of woven meshes: plain weave, twilled weave, plain Dutch weave, and twilled Dutch weave, all having periodic structure which makes them excellent base for SERS platform. They are used as a filtration media in chemical industry [43,44] due to their ability to withstand large pressures, high heat resistance, small aperture and reusability. We used twilled Dutch SSWM as a base for our SERS-platform: the SSWM was cleaned and sputtered with 50 nm thick layer of silver which ensured the high amplification of the Raman signal. The dense mesh structure also provided good deposition of the analyte on the surface.

The developed SERS platforms, named Ag/SSWM, show very high surface-enhancement factor (1 × 10^6^), high stability (up to one month under ambient conditions), high reproducibility, and high thermal resistance to laser irradiation. These Ag/SSWM substrates demonstrated a great potential in sensitive and reproducible SERS-based detection of typical analyte like *p*-MBA, as well as whole microorganisms, that is, *Escherichia coli* and *Bacillus subtilis.*

## 2. Materials and Methods

### 2.1. Chemicals and Materials

*P*-mercaptobenzoic acid (*p*-MBA) and phosphate-buffered saline (PBS) packs (10 mM, pH = 7.2) were obtained from Sigma-Aldrich (Dorset, UK) and used without further purification. Water (resistivity over 18 MΩ), purified using a Milli-Q plus 185 system was used throughout the process. Stainless steel wire mesh was obtained with Anping County Huijin Wire Mesh Co., Ltd., Anping, China. Each type of mesh wire was purchased in quantity of 1 m^2^ and deposited rolled in room temperature prior to use.

### 2.2. Instrumentation

#### 2.2.1. Raman and SERS Spectroscopy 

Measurements were carried out with a Renishaw inVia Raman system (Wotton-under-Edge, Gloucestershire, UK) equipped with a 785 nm diode laser (Wotton-under-Edge, Gloucestershire, UK)). The light from the laser was passed through a line filter and focused on a sample mounted on an X–Y–Z translation stage with a 50× microscope objective, Numerical Aperture (NA) = 0.75. The beam diameter was approximately 5 µm. The laser power at the sample was 5 mW or less. The microscope was equipped with 1200 grooves per mm grating, cut-off optical filters, and a 1024 × 256 pixel Peltier-cooled RenCam CCD detector (Wotton-under-Edge, Gloucestershire, UK), which allowed registering the Stokes part of Raman spectra with 5–6 cm^−1^ spectral resolution and 2 cm^−1^ wavenumber accuracy. The experiments were performed at ambient conditions using a back-scattering geometry. The time required for completing a single SERS spectrum was 4 seconds for *p*-MBA and 40 seconds for bacteria.

The obtained spectra were processed with an OPUS software (Bruker Optic GmbH 2012 version, Ettlingen, Germany). The spectra were smoothed with Savitsky-Golay filter, the background was removed using baseline correction, and then the spectra were normalized using a so-called Min-Max normalization.

#### 2.2.2. Scanning Electron Microscopy (SEM) 

Observations were performed under high vacuum using the FEI Nova NanoSEM 450 (Hillsboro, OR, USA). The accelerating voltage was in rage from 2 to 10 kV. The SSWM samples with bacteria cells were observed without any additional layer of gold.

### 2.3. Preparation of the SERS Platform and Sample Measurement

**Figure 1:**
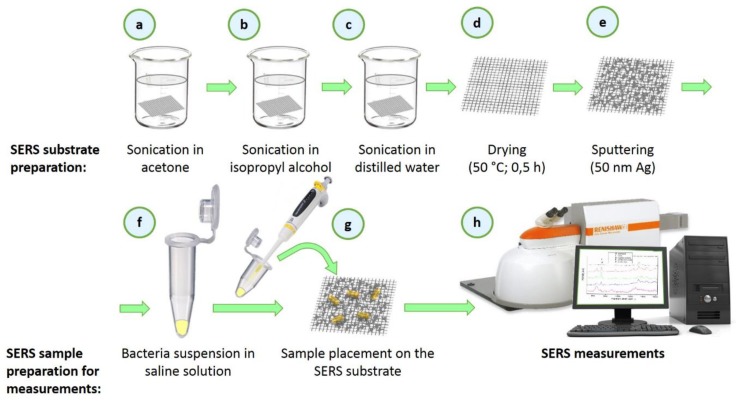
The scheme showing the preparation of SERS substrate, sample deposition and SERS measurement. The main steps involve cleaning (**a**–**c**), drying (**d**), sputtering of thin layer of silver (**e**), deposition of bacteria on SSWM platform (**f**,**g**), and SERS measurement (**h**).

General scheme of preparation of SERS-active platform for Raman measurements is shown in Figure 1. Wire mesh sample (40 × 40 mm) was cut with scissors from big sheet (100 cm × 100 cm) and placed in baker filled with acetone. In the first step the sample was sonicated for 10 min in ultrasonic bath at a temperature of 50 °C (Figure 1a). Then the acetone was exchanged and the step was repeated. After 10 min the baker was filled with isopropyl alcohol and the sample was sonicated for 10 min at a temperature of 50 °C (Figure 1b). Then the sample was sonicated for 10 min in distilled water (Millipore) at ambient temperature (Figure 1c). Cleaned wire-mesh was then dried for 30 min at 50 °C (Figure 1d) and placed in a sterile Petri dish or immediately placed in a Physical Vapour Deposition (PVD) device and sputtered with a 50 nm layer of silver (Figure 1e). The prepared SERS platform is ready for use.

To perform our measurements, we placed three bacterial colonies in micro-tubes filled with 500 µL of saline solution (Figure 1f), which was vortexed and then placed on platform with pipette (Figure 1g). Then the SERS platform with deposited bacteria was placed under microscope and subjected to measurements (Figure 1h).

### 2.4. Bacteria CCulture and its Preparation for SERS Measurements

#### 2.4.1. Bacteria Culture and SERS Sample Preparation

*E. coli* and *B. subtilis* were obtained from the Department of Applied Microbiology, Institute of Microbiology, University of Warsaw, Warsaw, Poland. In the case of each species, bacteria were streaked on the Petri dishes with LB agar (Luria-Bertani broth agar) and incubated at 37 °C for 24 h. Next, three single bacterial colonies were suspended in 500 µL of saline solution (0.9% NaCl solution) and centrifuged for 5 min at 1070× *g*. Then, the supernatant was decanted and the pellet of bacterial cells was re-suspended in 500 µL of saline solution. The obtained concentration of bacterial cells was 5 × 10^9^ mL^−1^. This process was repeated three times in order to obtain clean sample of microbial cells without any additional contamination form cell culture medium. After discarding the supernatant in the last step of centrifugation, bacteria cells were suspended in 20 µL of saline solution, transferred via pipette and placed onto SSWM substrate (Figure 1f,g). The sample was left to dry for 5 min and then the SERS measurements (Figure 1h) were conducted.

#### 2.4.2. Procedure of Silver Sputtering

To sputter a layer of silver we used the PVD equipment (EM MED020, Leica, Heerbrugg, Switzerland). The silver target was obtained from Mennica Metale Szlachetne, Warsaw, Poland. The target diameter was 54 mm, thickness was 0.5 mm, and silver purity was 4 N. The vacuum during the silver sputtering was on the level of 10^−2^ mbar, whereas the sputtering current was 25 mA. After the deposition process the samples were placed into a sterile Petri dish. 

Six different thicknesses of silver layer on the SSWM substrates, i.e., 5, 20, 35, 50, 70 and 100 nm, were tested to find optimal conditions for SERS enhancement. 

## 3. Results and Discussion

### 3.1. Characterization of Wire Mesh and its Surface

In our experiments, we used five different wire meshes (see Table 1), made of stainless steel 316 (austenitic chromium-nickel stainless steel containing molybdenum). Addition of molybdenum increases general corrosion resistance, improves resistance to pitting from chloride ion solutions, and provides increased strength at elevated temperatures. The corrosion resistance of 316 steel is improved, particularly against acid sulphates, alkaline chlorides, and sulfuric, hydrochloric, acetic, formic and tartaric acids. What is important, the thermal conductivity of steel 316 is 16.3 W·m^−1^·K^−1^, whereas polymer, for example, PLA thermal conductivity is ca. 0.19 W·m^−1^·K^−1^ [45]. That large thermal conductivity of stainless steel is what makes it superior base material for SERS platform.

**Figure 2:**
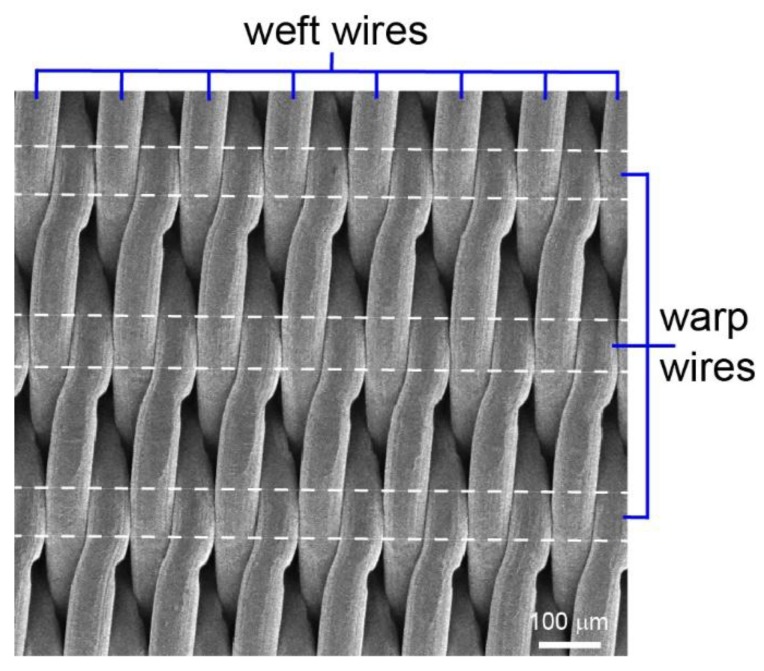
Twill Dutch weave is a densly woven fabric made of 316 stainless steel. It consists of two types of wires: warp (with a higher diameter) and weft (with a smaller diameter). The presented picture is a SEM of 80 × 800 wire mesh.

**Figure 3:**
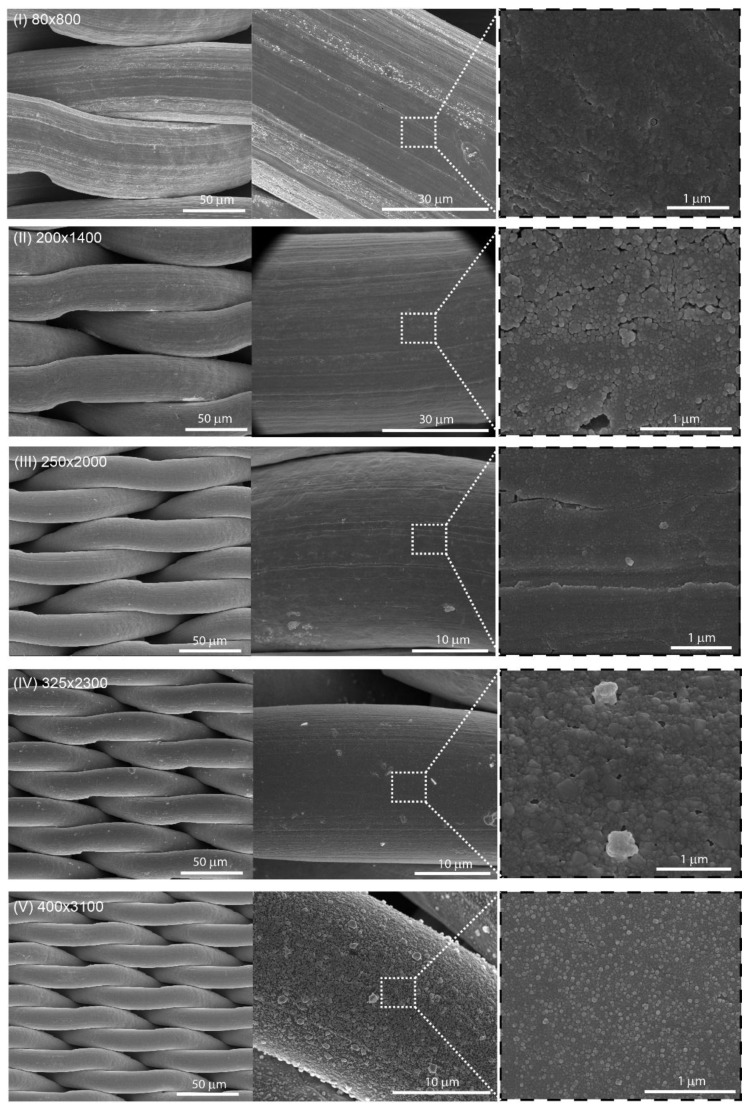
SEM images of SERS-active platforms (sputtered with 50 nm layer of silver via PVD technique) at three different magnifications. Parameters of the wire meshes are described in Table 1.

Figure 2 presents general organization of the wires in twill Dutch weave fabric, while Figure 3 presents SEM pictures of all wires meshes (see Table 1) at three different magnifications. As can be seen, the twill Dutch type is extremely dense and tightly woven fabric. Twill refers to the structure of the fabric: over two and under two weaving wires with respect to the warp wires (warp are wires running lengthwise of the cloth, whereas weft are wires running across the cloth). The term Dutch means that the weft wires have smaller diameter than the warp wires (see Table 1 for details). The structure of twill Dutch weave is presented in Figure 3, whereas the schematic illustration of the weft and warp wires structure is presented in Figure S1.

Twill Dutch weave enables the weft wires to be woven more densely. Therefore, much smaller aperture sizes can be achieved. The term mesh (e.g. 80 × 800) refers to the number of warp (80) and weft (800) wires per inch. 

Figure 3 shows the representative SEM images of five wire meshes (I–V), for three different magnifications. All samples were sputtered with 50 nm of silver via PVD method as described before. This method provides a homogeneous coverage of the SSWM surface with the layer of silver. 

**Figure 4:**
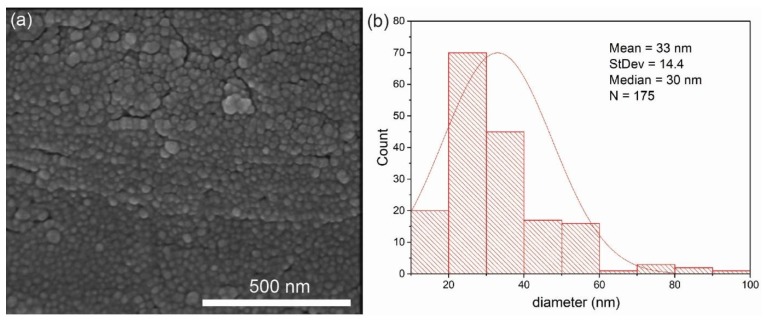
The surface of 80 × 800 (I) wire mesh covered with 50 nm layer of silver (**a**) and histogram of the size of the Ag nanostructures on the surface (**b**). The avarage size of these nanostructures is 33.0 nm ± 14.4 nm.

SEM image at high magnification exhibits homogeneously placed silver nanostructures. The image analysis has been performed to quantify their size distribution. Representative image and its histogram for sample I (80 × 800) is presented in Figure 4, while the histograms for all studied samples are depicted in Figure S2. It can be noticed that for all samples the avarage size of the silver nanostructures on the surface of the wires is below 45 nm, while the median is below 40 nm. These parameters are optimal for the LSPR.

### 3.2. SERS Properties of Ag/SSWM Substrate

The SERS efficiency (sensitivity, selectivity, and reproducibility) is strongly correlated with the morphology of the SERS-active nanostructures. The *p*-MBA was used as a standard probe compound in order to prove the SERS properties of fabricated Ag/SSWM substrates. In this study, five different wire mesh surfaces (Figure 3) have been investigated in the terms of their spectroscopic properties.

In the first step, the SERS sensitivity of the Ag/SSWM substrates was examined and described by the enhancement factors (EF) for *p*-MBA. The *p*-MBA molecules bind through its thiol groups to the top silver layer of SERS-active surface and thus allow to record the intense SERS responses. Moreover, the intensities of *p*-MBA SERS features depend on the plasmonic properties of the SERS substrate and are not affected by the possible electronic resonance mechanism.

The Ag/SSWM substrate was kept in 1.0 mL of 1.0 × 10^−6^ M *p*-MBA ethanol solution for 3 h and then washed with deionized water. The Raman bands at 708, 796, 1075, 1176, 1474 and 1588 cm^−1^ are characteristic for *p*-MBA [46]. The surface enhancement factors for *p*-MBA have been calculated according to the standard equation:

(1) $$EF=\frac{I_{SERS}N_{NR}}{I_{NR}N_{SERS}}$$

where *N*_NR_ stands for the number of molecules sampled by normal Raman measurements, whereas *N*_SERS_ describes the number of molecules irradiated in SERS technique. *I*_NR_ and *I*_SERS_ were measured at 1075 cm^-1^ and correspond to the normal Raman scattering intensity of *p*-MBA in the bulk and the SERS intensity of *p*-MBA adsorbed to metallic nanostructures. 

The laser spot area and the effective illuminated volume are fundamental parameters for the estimation of EF. The effective illuminated volume has been calculated using a formula recommended by Renishaw:

*V* = 3.21 × λ^3^(*f*/D) (2)

where *f* is the microscope objective focal length and D represents the effective laser beam diameter at the objective back aperture. For our setup, *V* = 2012 ≈ 2×10^3^ µm^3^. The laser beam diameter, defined as twice the radius of a circle encompassing the area with 86% of the total power was about 2.5 µm. It should be highlighted that approximately the same values were achieved from the experimentally obtained laser spot image and from the theoretical formula (4λ*f*/πD). Assuming the volume in a shape of a cylinder with the diameter of 5 µm leads to the effective height of 100 µm. This value was confirmed by recording Raman spectra of Si while varying the distance from the focal plane. 

The SERS samples were prepared by immersing the substrate in 1 mL of 1.0 × 10^−6^ M solution of *p*-MBA. The number of molecules in the solution was 6.02 × 10^14^ (6.02 × 10^23^ molecules/mol × 1.0 ×10^−3^ L × 1.0 × 10^−6^ mol/L = 6.02 × 10^14^ molecules). The surface area irradiated by the laser beam (2.5 µm in diameter) was 19.6 µm^2^ (3.14 × 1.25 μm^2^ = 4.9 µm^2^). The surface of our samples was 10 mm^2^. Therefore, about 2.3 × 10^8^ molecules were present in the laser beam spot. The normal Raman spectrum was observed for a cell filled with a pure *p*-MBA (154.19 g·mol^−1^; density of 1.06 g·cm^−3^). The effective illuminated volume for our setup is 2 × 10^3^ µm^3^. Under these conditions, *N*_NR_ = 8.1 × 10^12^ molecules were irradiated by the laser. From these data of the relative intensity and the number of molecules sampled from the regular Raman and SERS measurements, the enhancement factors for all studied SERS surfaces have been calculated.

**Figure 5:**
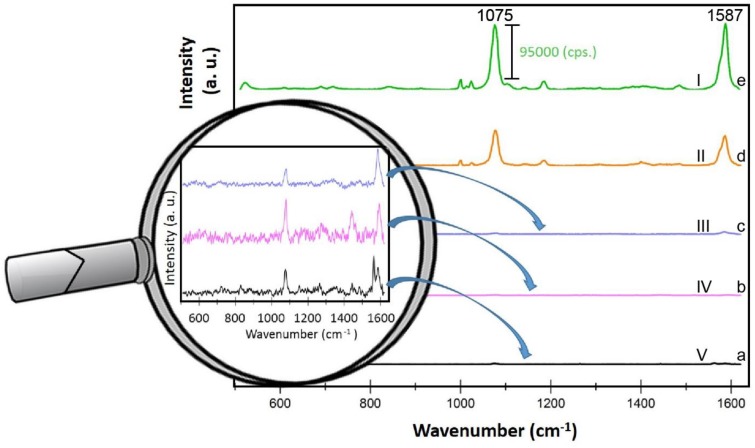
SERS spectra of *p*-MBA recorded from five different SERS substrates (a-e) with varying morphology of wire mesh (according to parameters described in Table 1). Experimental conditions: 5 mW of 785 nm excitation, 2 × 5 seconds acquisition time. The image in magnifier presents the close view of the region with marker band at 1075 cm^−1^ for SSWM surfaces showing low EF. Each SERS spectrum was averaged from twenty measurements in different places of SERS surface.

Figure 5 depicts the SERS spectra of *p*-MBA molecules adsorbed from 10^−6^ M ethanol solution onto five studied surfaces (Figure 3). The enhancement factors for each surface (named from I to V) were calculated using equation (1) and are presented in Table 2. The highest enhancement factor has been found for the surface I (Table 2), which indicate that its morphology matches to the optimal size of nanostructures (20–40 nm) for the efficient LSPR. 

**Figure 6:**
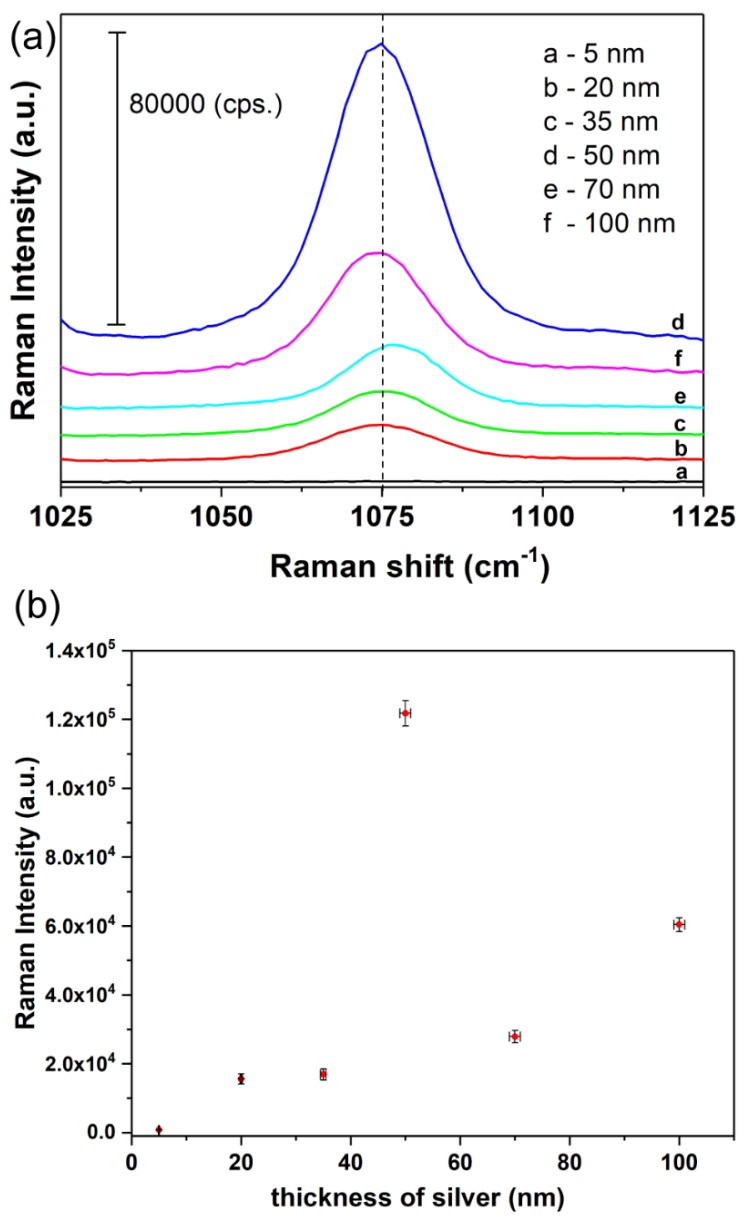
The intensity of SERS band at 1075 cm^−1^ with varying Ag metal thickness (5 nm, 20 nm, 35 nm, 50 nm, 70 nm and 100 nm) for Type I surface: mesh 80 × 800: (**a**) Raman spectra, (**b**) numerical values of Raman intensity. The error bars in the Figure 6b shows standard deviation of both thickness of the silver layer and Raman signal intensity.

As was mentioned above, in order to acquire SERS activity of the SSWMs platforms, they were covered with silver layer in PVD process. The deposited silver film thickness affects the size and the density of formed silver nanostructures [47]. Figure 6 shows the dependence between the intensity of the marker bands of *p*-MBA at 1075 cm^−1^ and the thickness of the deposited silver film. As can be seen the 5 nm layer of silver was insufficient to achieve the reasonable SERS responses for *p*-MBA. The intensity of *p*-MBA SERS spectrum increased significantly when the thickness of silver layer increased from 20 to 50 nm (Figure 6).

The maximum intensity of the marker bands of *p*-MBA has been recorded for 50 nm thickness of the deposited layers of silver. Further increase in the amount of silver leads to decrease in intensity of SERS bands.

It should be highlighted that, despite the numerous theoretical and experimental works, the nature of the Raman signal enhancement in SERS technique is still not obvious. However, as mentioned above, two enhancement mechanism (long-range electromagnetic effect (EM) and short-range charge–transfer effect) are involved in SERS phenomenon. It has been found that the dominating electromagnetic enhancement in SERS is caused by surface plasmon resonances on the substrate. This is an example of amplification of incident light intensity by excitation of surface plasmons. Briefly, the factors which determine the absorbance and the bandwidth of the plasmon resonance include the size, shape, and density of the metal nanostructures. In this case the morphology and microstructure of the Ag nanofilms onto SSWM also play a crucial role in EM effect of SERS enhancement.

These results indicate, that the 50 nm thickness of the Ag metal film results in an optimal size (average size of the objects is 33.0 nm ± 14.4 nm) and distribution of silver nanostructures for a plasmon resonance effect for 785 nm laser line. The distribution of the nanostructures determines the formation of ‘hot spots’ on the SERS substrate. It is possible that mentioned factors (suitable size of silver nanostructures, number and distribution of ‘hot spots’) make significant contribution to the observed SERS enhancement.

The thicker layers of silver (70 and 100 nm) results in decrease of SSWM roughness and thus lead to the loss of optimal condition for LSPR [48]. 

To summarize: (i) the stainless steel wire Type I, mesh 80 × 800 (Table 1, Figure 3) and (ii) 50 nm thickness of the Ag metal film results in obtaining the most efficient SERS-active platform. This type of substrate has been applied for further analysis of bacterial cells. In all experiments the 785 nm excitation wavelength was applied, as it is the compromise between the signal intensity and background fluorescence [49], especially during the measurements of biological samples. Additionally, we have also checked that the SERS intensity of 1075 cm^−1^ marker band of *p*-MBA increases ranging from 532 to 785 nm (see Table S1 in Supplementary Materials). This behavior has been recently discussed in literature [50]. The reflectance spectra of enhancing substrates are used to the detection of far-field electromagnetic (EM) enhancement mechanisms, while the near-field modes could be responsible of the strong long-wavelength resonances in the range of 700−850 nm [50], and explain these experimental results. The dark plasmons (example of non-radiating modes) detected in nanogaps and/or aggregated nanostructures [51] are indicated as potential origins of such huge Raman signal enhancements [52].

It should be highlighted that the developed Type I SERS-active surface show excellent sensitivities for *p*-MBA, also at concentrations as low as 10^−9^ M. Figure S3 depicts the SERS spectra of *p*-MBA adsorbed onto Type I SERS surface at different concentrations (a) 10^−3^ M, (b) 10^−6^ M, and (c) 10^−9^ M in ethanol. It is clear that a steady decrease in SERS intensity of the *p*-MBA SERS band is observed with decreasing *p*-MBA concentration. However, even at 10^−9^ M concentration the intensity of the marker band at 1075 cm^−1^ is above 1000 cps for 2 × 2 seconds of acquisition time, which still enables the SERS sensing.

### 3.3. Applications of Ag/SSWM SERS Substrate: Differentiation Between Gram-Positive and Gram-Negative Bacteria Species

**Figure 7:**
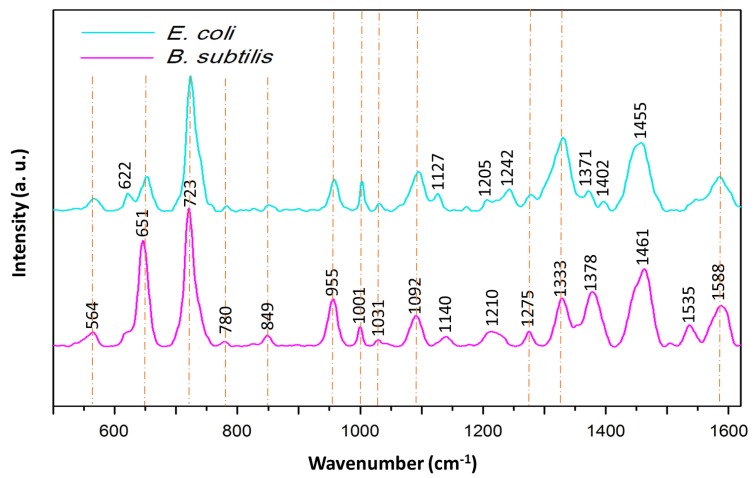
Average SERS spectra *E. coli* (turquoise) and *B. subtilis* (pink) recorded on Ag/SSWM (mesh 80 × 800 covered with 50 nm layer of Ag) SERS platforms. For all spectra, excitation wavelength was at 785 nm, laser power was 5 mW, and acquisition time was 30 seconds. Each SERS spectrum of examined bacteria was averaged from 30 measurements in different places across the SERS surface using mapping mode.

In order to present the bioanalytical potential of developed SERS surface the SERS spectra of two different bacteria species: *E. coli* (Gram-negative) and *B. subtilis* (Gram-positive) were recorded. As can be seen in the Figure 7, the obtained SERS spectra of both bacteria species show major peaks at around 650, 725, 960, 1000, 1100, 1330, 1375, 1460 and 1590 cm^−1^ and less intense bands at around 565, 780, 850, 1030, 1275 cm^−1^. The peak at around 565 cm^−1^ may originate from *C–O–C* ring deformation [53] or *C–C* skeletal vibration [54], while the one at 650 cm^−1^—from *C–S* stretching in methionine [55] and/or *C–C* twisting mode of tyrosine [56]. The most intense band, located at 725 cm^−1^, can be assigned to adenine derivatives [57]. The peak at 780 cm^−1^ comes probably from ring breathing modes in the DNA/RNA bases (uracil, thymine and cytosine) [58] and the one at 850 cm^−1^—from asymmetric *O–P–O* stretching and/or tyrosine [59]. The bands at ~960 and 1000 cm^−1^ originate probably from *C–N* stretching [60] and from phenylalanine [61], respectively. The phosphodioxy group ($PO_{2}^{-}$) from nucleic acids can be observed in the spectrum in a form of the peak at 1092 cm^−1^ [62], while the amide III and *CH_2_* wagging vibrations from glycine backbone and proline sidechains can be detected as the band at 1275 cm^−1^ [63]. Finally, the bands at around 1333, 1375, 1460 and 1590 cm^−1^ can be assigned to adenine [64], *COO* stretching [65], *CH_2_* bending [53] and *C=C* olefinic stretching [60], respectively.

Although the presented in Figure 7 spectra show a lot of similar bands, there still can be noticed some peaks due to which the obtained spectra can be easily distinguished. This refers to the bands at 620, 1127, 1205, 1242 and 1402 cm^−1^ which can be seen only in the case of *E. coli* spectrum, and to bands located at 1140 and 1535 cm^−1^, which are detected only in *B. subtilis* spectrum. Moreover, some SERS signals, which are present in the spectra of both bacteria species, are more intense for *E. coli*, for example, 1001 and 1333 cm^−1^, and some for *B. subtilis*, for example, 650 and ~1375 cm^−1^. Based on these differences it can be concluded that Ag/SSWM SERS substrates can be applied for successful detection and identification of bacteria.

**Figure 8:**
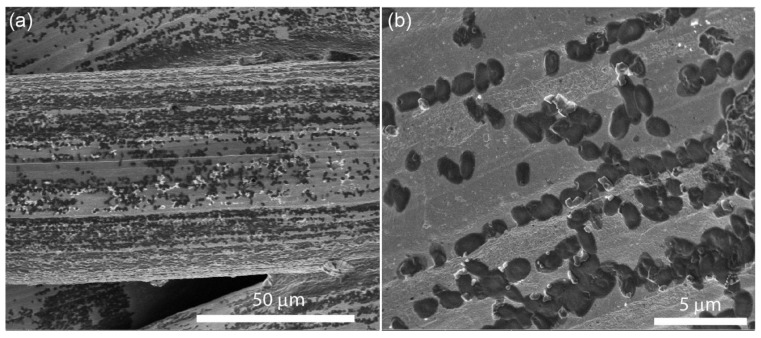
SEM images of *E. coli* placed onto Ag/SSWM surface and collected at (**a**) lower and (**b**) higher magnification. The bacteria are arranged in lines due to irregularities of wire surface.

Additionally, the presence of bacteria cells onto the Ag-coated SSWM have been confirmed by SEM images (see Figure 8). The images show the *E. coli* cells, approximately 2–2.5 µm in diameter, scattered uniformly over the steel wire. Figure 8b reveals that bacteria are arranged in lines. The reason of such arrangement is connected with the structure of the stainless wire, which is produced *via* wire drawing. This process produces unevenness on the wire surface, which are parallel to the wire axis. These irregularities (scratches) are in the scale of single micrometres and perfectly match the diameter of bacteria cells. 

### 3.4. Reproducibility and Thermal Resistance of the SERS Substrate

**Figure 9:**
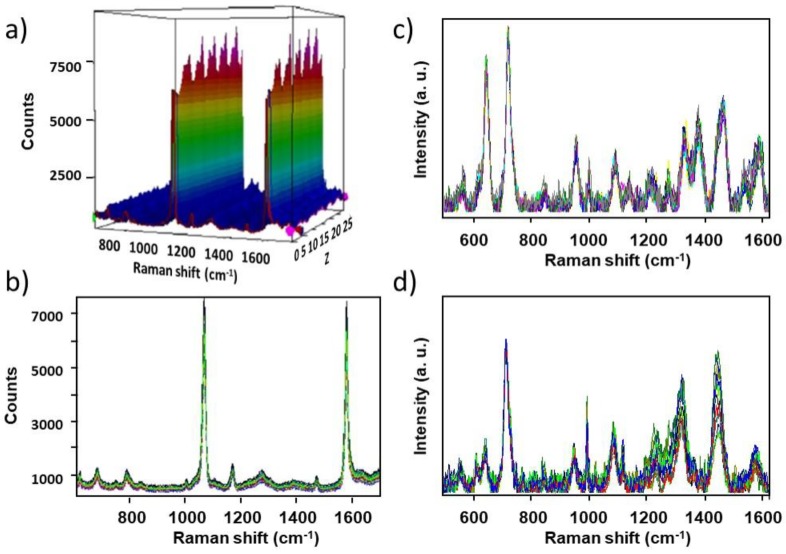
The representative SERS spectra of (**a**,**b**) *p*-MBA of concentration of 10^-6^ M, (**c**) *B. subtilis*, and (**d**) *E. coli* recorded from 30 different spots on the SERS surface (Type I) using mapping mode. The spectra were collected over a distance of 1 mm with 10 µm steps (30 spectra are shown). Each point in the map was recorded using 5 mW of 785 nm excitation.

To estimate the reproducibility of prepared Ag/SSWM substrates, the relative standard deviation (RSD) was calculated for SERS spectra of *p*-MBA, *B. subtilis*, and *E. coli.* Figure 9 and Figure S4 presents obtained results for particular Type I substrate. The calculated RSD values equal 6.4%, 11%, and 9.5%, respectively. All these data indicate that the developed SERS substrates can be applied in biomedical and analytical studies. 

The application of SERS technique within biology and medicine is a rapidly expanding field of science since SERS can provide chemical, biochemical, and structural information through the generation of fingerprint spectra or spectral imaging. This method of analysis does not require a complex sample preparation, and can also be applied without a complicated labelling strategy, and does not suffer from interference from water. However, besides the long list of advantages there are some limitations associated with SERS techniques. The wavelength of the exciting laser and its power onto the irradiated sample are the key factors that determine the spectral resolution. The spatial resolution is defined by the optic of microscope objective and the wavelength of the laser. In biological studies the NIR (near-infrared) lasers at 785 nm and 830 nm, which have relatively low photon energy and allow the reduction of fluorescence contribution in Raman spectrum, are commonly used. The power of laser illuminating the sample depends on the laser spot size and the magnification of the microscope. His in turn results in the intensity of scattered light. The biological samples are usually low-scattering materials and are very sensitive to the radiation damage or local thermal decomposition. 

The burning or photo-degradation of biological samples, especially over prolonged period of excitation at too high laser power often results in appearance of the additional band in the recorded SERS spectrum at ca. 1500 cm^−1^ (Figure S5). The presence of the mentioned peak is usually associated with the formation of amorphous carbon. On the other hand, the reduced laser power during measurements results in very poor spectral quality and generate an invaluable information. 

To detect such week intensities of Raman scattered light and to obtain high-quality spectral features the special thermal resistant SERS-active substrates—which enable effective distribution and/or heat dissipation during intensive laser illumination—have been applied. These thermal resistant SERS-active platforms can be used not only for detection/identification of biological specimens, but also for examination of variety of thermally degradable analytes or materials. 

**Figure 10:**
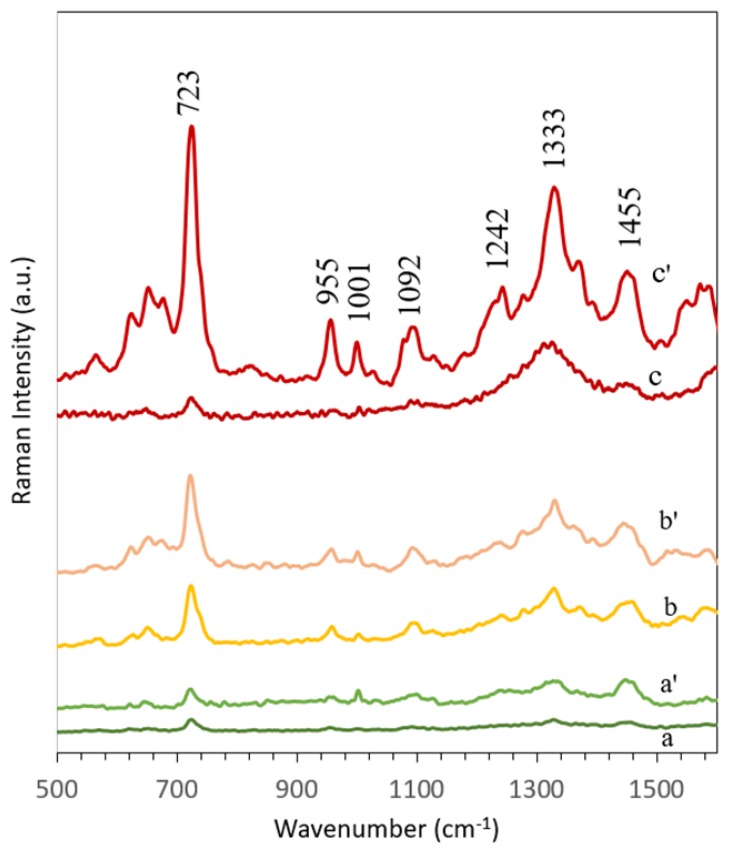
The SERS spectra of *E. coli* recorded on two different SERS substrates based on: polymer mat —PLLA (a, b, c) and the stainless steel wire mesh – SSWM (a^’^, b^’^, c^’^) recorded with different powers of 785 nm excitation wavelength: (a, a^’^) 0.6 mW, (b, b^’^) 1.3 mW, and (c, c^’^) 14.5 mW.

To demonstrate the thermal capability of developed SERS-active surfaces the same volume of *E. coli* in saline solution has been deposited onto two different kinds of the SERS substrates based on: (1) poly-L-lactic acid (PLLA) and (2) SSWM surfaces. The SERS data have been recorded at various intensities of excited laser power (see Figure 10). 

The results demonstrate that 14.5 mW power of laser degrades *E. coli* on PLLA polymer mat/Ag, whereas bacteria on SSWM/Ag exhibit excellent spectra. Such result can be explained by very good thermal conductance of stainless steel (nearly 100 times higher than for PLLA). Therefore, no significant increase in temperature at the measurement spot is observed in the case of SSWM. In practice, the laser power below 5 mW is used for SERS measurements of biological samples. 

To summarize, the results presented in this work show an excellent sensitivity, reproducibility, and thermal stability of obtained SERS substrates (Type I), which can be successfully used during the detection of biological samples.

## 4. Conclusions

The present study demonstrates a new type of SERS platform based on woven wire mesh made of stainless steel (SSWM). The influence of the diameter of wires, their morphology and metal thickness on SERS efficiency was investigated and the optimal fabrication process parameters were established. In this paper, for the first time, we present the SERS substrate that exhibits excellent heat dissipation, which is a critical factor in SERS measurements of thermally sensitive samples. Our substrate enables generation of the high spectral resolution data even for very poorly scattering biological materials *via* safe adjustment of laser intensity to higher values. This approach overcomes the limitations associated with photo-and thermal degradation of sensitive materials, improves the spectral intensities of week Raman scatterers, and thus extends the rage of valuable SERS applications. In addition, the developed SERS substrate enables the detection and differentiation of Gram-positive and Gram-negative bacterial species in label-free manner, based on their high-quality spectral features.

## Figures and Tables

**Table 1:** Types and parameters of wire meshes used in experiments. All wire meshes are made of stainless steel 316. Mesh refers to the number of warp and weft wires per inch.

Number of Sample	Mesh	Warp Diameter [µm]	Weft Diameter [µm]	Type of Weave
I	80 × 800	120	70	twill Dutch
II	200 × 1400	50	40	twill Dutch
III	250 × 2000	45	27	twill Dutch
IV	325 × 2300	35	25	twill Dutch
V	400 × 3100	30	17	twill Dutch

**Table 2:** The EF factors for five different Ag/SSWM substrates (Table 1).

Number	Mesh	Enhancement Factor (EF)
I	80 × 800	4.2 × 10^6^
II	200 × 1400	1.3 × 10^5^
III	250 × 2000	1.0 × 10^3^
IV	325 × 2300	1.2 × 10^3^
V	400 × 3100	0.8 × 10^3^

## Supplementary Materials

The following are available online at http://www.mdpi.com/2079-4991/8/9/663/s1.
