# Supplementary material for: Steel Wire Mesh as a Thermally Resistant SERS Substrate
Source: Nanomaterials (Basel). 2018 Aug 26;8(9):663. doi: 10.3390/nano8090663 (PMC6163328; doi:10.3390/nano8090663)
Supplement: Supplementary file 1 [file nanomaterials-08-00663-s001.pdf]

Supplementary Materials

Steel mesh wire as a thermally resistant SERS platform

T. Szymborski[a,b], E. Witkowska[a], K. Niciński[a], Z. Majka[a], T. Krehlik[a], K. Winkler[a] and A. Kamińska[a]*

[a]Institute of Physical Chemistry, Polish Academy of Sciences, Kasprzaka 44/52, 01-224 Warsaw, Poland

[b]Soft Materials Laboratory, Institute of Materials, Ecole Polytechnique Fédérale de Lausanne, 1015 Lausanne, Switzerland

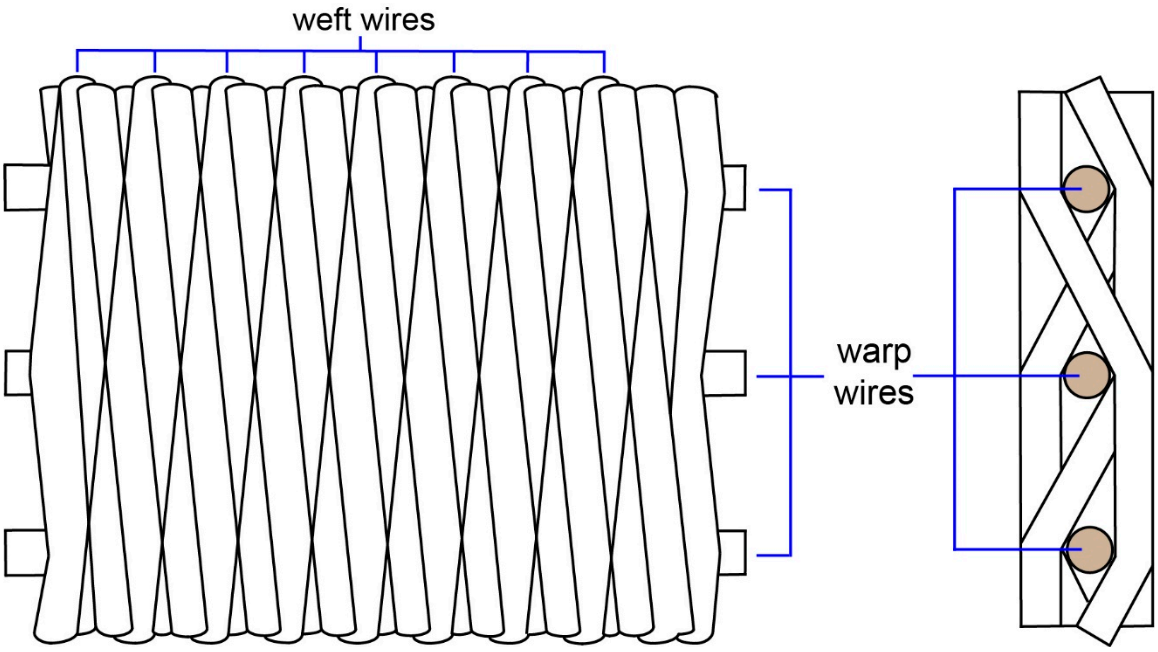

Figure S1. The twill dutch woven consists of two perpendicular types of wires: weft and warp. The warp has higher diameter than weft (in case of 80×800, the warp has 120 µm and the weft 70 µm). The term till refers to the structure of the fabric: over two and under two weaving wires with respect to the warp wires (see left figure for details).

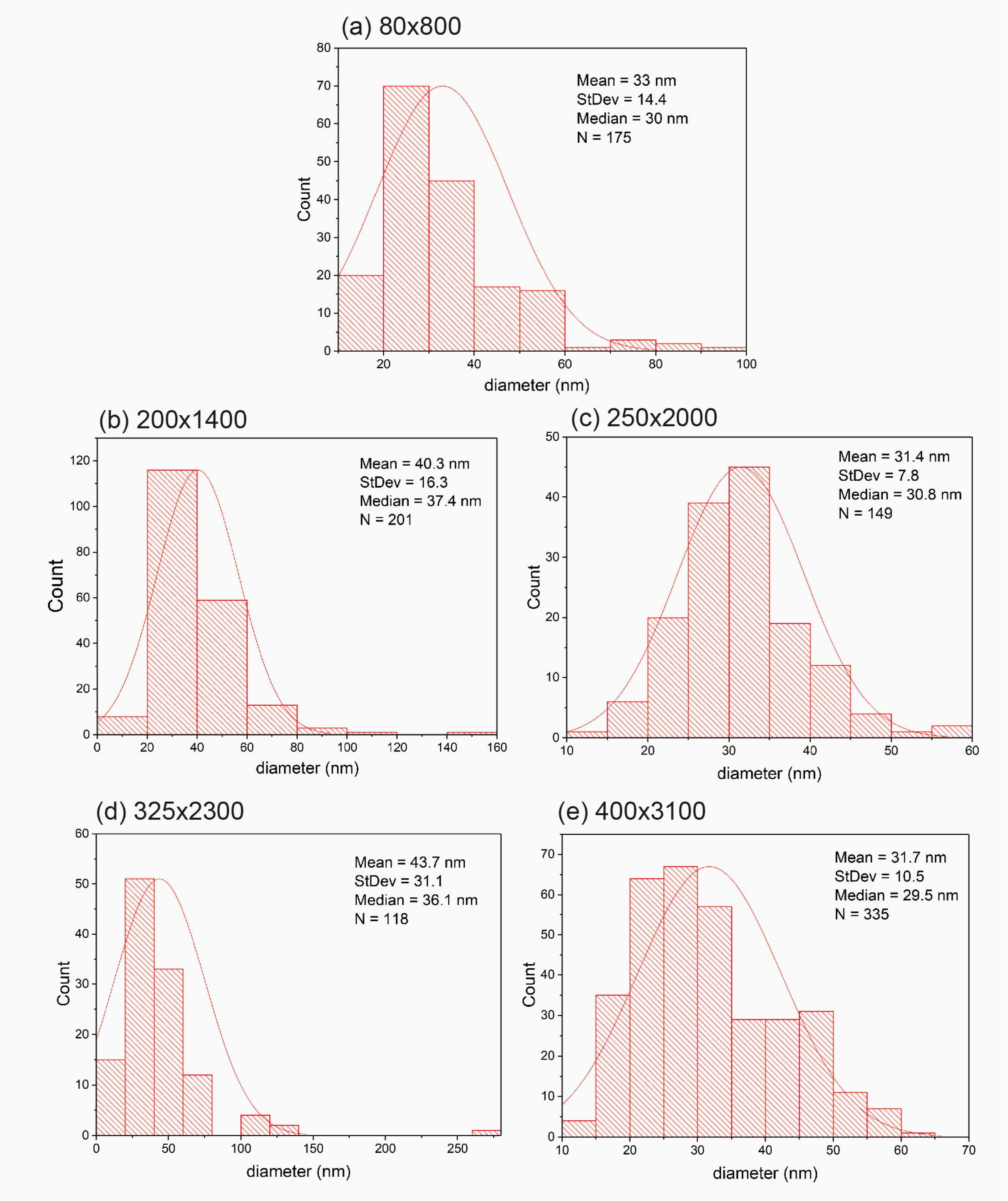

Figure S2. Histograms of the size of the silver objects on the surface of the mesh wire.

Excitation Wavelength (nm)	Intensity of the marker band of *p*-MBA at 1075 cm^{-1} (cps, counts per second)
532	4500
632.5	98000
785	12000

Table S1. The intensities of the marker band at 1075cm-[1] of the representative SERS spectra of *p*-MBA adsorbed onto Ag/SSWM substrates collected at the three excitation wavelengths 532, 632.8, and 725 nm, respectively.

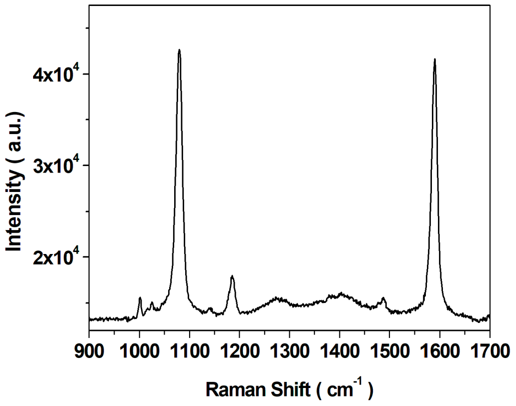

a

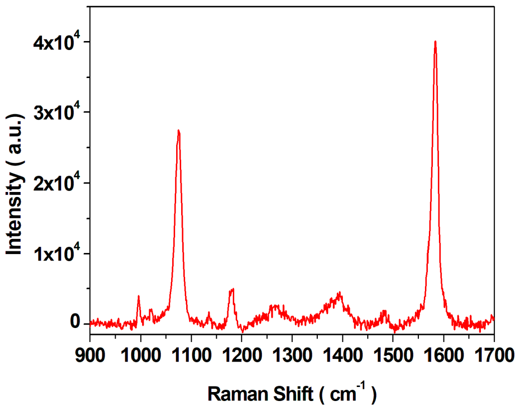

b

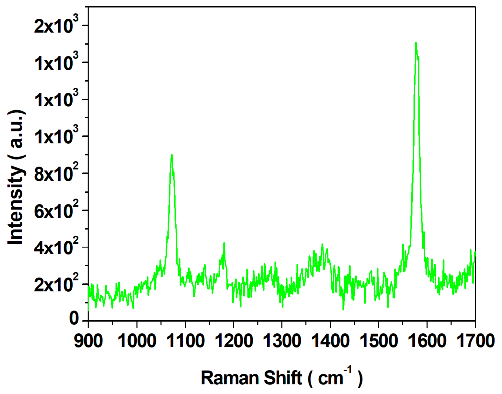

c

Fig. S3. The SERS spectra of *p*-MBA adsorbed onto " Type I" SERS surface at different concentration (a) 10^{-3}M, (b) 10^{-6}, and (c) 10^{-9} M in ethanol.

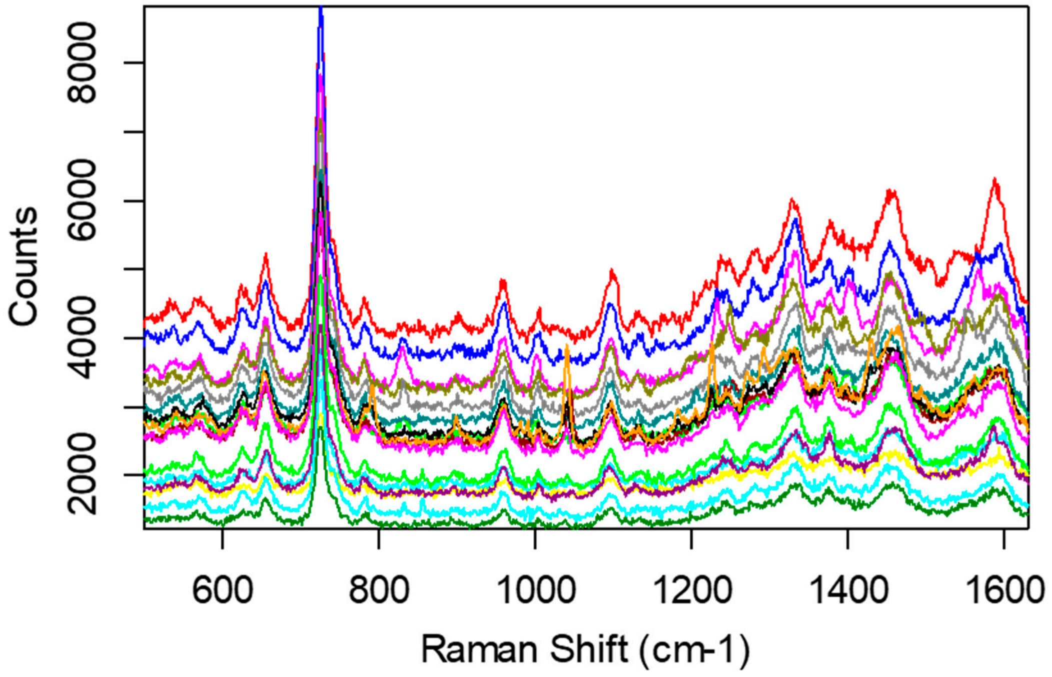

Figure S4. SERS spectra of *E. Coli* recorded from different points across the SERS substrate.

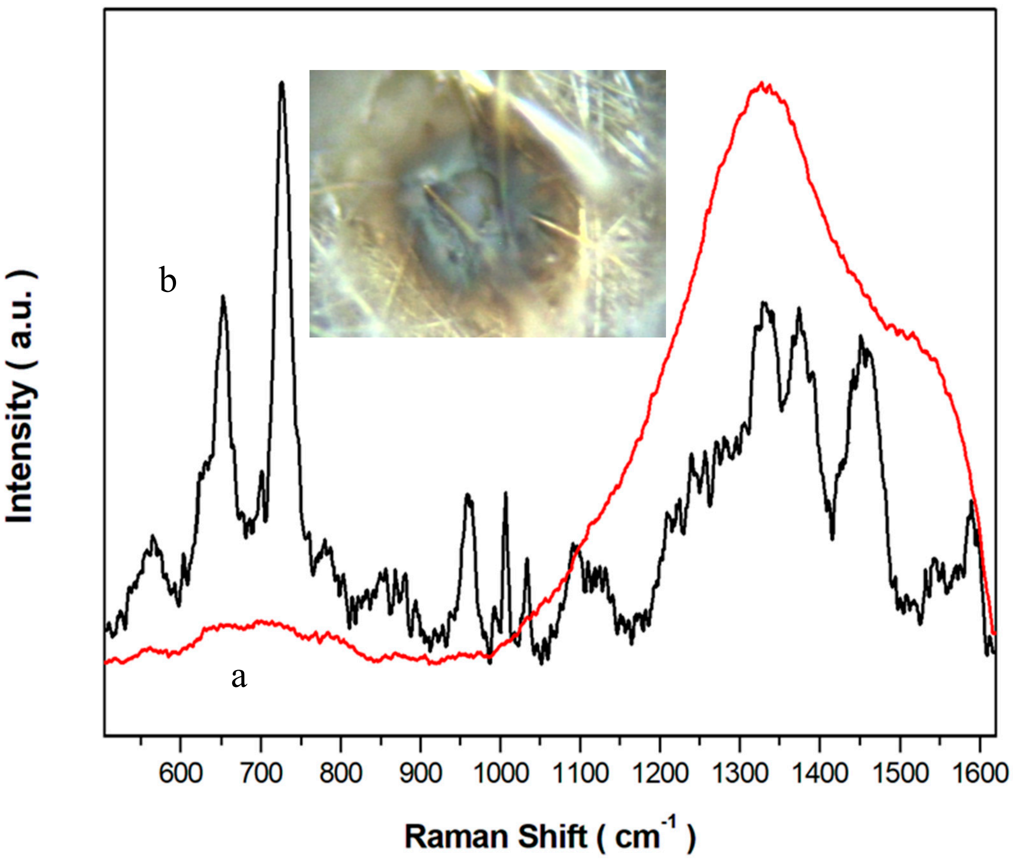

Fig. S5. The SERS spectra of *E. coli* recorded onto polymer mat – PLLA at different power of the 785 nm excitation wavelength: (a) 1.3 mW, and (b) 14.5 mW**.** Insert presents the image of destroyed sample observed via optical microscope.
